# Angiopoietin-like 3 inhibition and the liver: less is more?

**DOI:** 10.1097/MOL.0000000000000898

**Published:** 2023-10-11

**Authors:** Reindert F. Oostveen, G. Kees Hovingh, Erik S.G. Stroes

**Affiliations:** Department of Vascular Medicine, Amsterdam Cardiovascular Sciences, Amsterdam UMC, University of Amsterdam, Amsterdam, The Netherlands

**Keywords:** antisense oligonucleotide, ARO-ANG3, liver safety, small interfering RNA, vupanorsen

## Abstract

**Purpose of review:**

The aim of this study was to discuss the potential mechanisms and implications of the opposing liver safety results from recent angiopoietin-like 3 (ANGPTL3) inhibition studies.

**Recent findings:**

The clinical development of vupanorsen, a N-acetylgalactosamine (GalNAc) antisense targeting hepatic ANGPTL3, was recently discontinued due to a significant signal of liver transaminase increase. Vupanorsen elicited a dose-dependent increase in hepatic fat fraction up to 75%, whereas the small interfering RNA (siRNA) ARO-ANG3, has reported preliminary evidence of a dose-dependent decrease in hepatic fat fraction up to 30%.

**Summary:**

ANGPTL3 inhibition is an attractive therapeutic target to reduce all apoB-containing lipoproteins. The discrepancy in liver signal results between the antisense and siRNA approach may be explained by the level of target inhibition. An alternative explanation may relate to off-target effects of vupanorsen, which have a molecule- and/or platform-specific origin. For intrahepatic strategies, highly potent ANGPTL3 inhibition will for now require special attention for liver safety.

## INTRODUCTION

Atherosclerotic cardiovascular disease (ASCVD) remains the leading cause of mortality worldwide [1]. Accumulation of apolipoprotein B containing lipoproteins, such as LDL cholesterol (LDL-C) and remnant-cholesterol derived from triglyceride-rich particles is a pivotal step in atherosclerosis [2]. Human genetic studies, prospective studies as well as randomized clinical trials have provided consistent evidence that apoB containing particles should be as low as possible for as long as possible in order to achieve the maximum benefit [3]. Guideline recommended LDL-C levels are not achieved in the vast majority of patients, despite the use of a large number of approved therapies to reduce apolipoprotein B containing lipoproteins [4]. The most commonly prescribed lipid lowering therapies such as statins, ezetimibe and PCSK9 inhibitors, are highly effective in reducing the apoB containing LDL-C particles, but their impact on apoB-containing triglyceride-rich lipoproteins is modest [5]. In addition, patients with severely reduced LDL-C receptor activity (mostly homozygous familial hypercholesterolemia) also have a large residual LDL-C burden reflecting the fact that the majority of agents available exert their effect via the LDL-C receptor clearance pathway [6]. Thus, there is an unmet medical need for novel lipid-lowering therapies, which should ideally have a large impact on both LDL-C and triglyceride-rich lipoproteins preferably acting via an LDL-C receptor independent pathway. Recently, angiopoietin-like 3 (ANGPTL3) has received a lot of attention as a promising therapeutic target to decrease all apolipoprotein-B containing lipoproteins. 

**Box 1:**
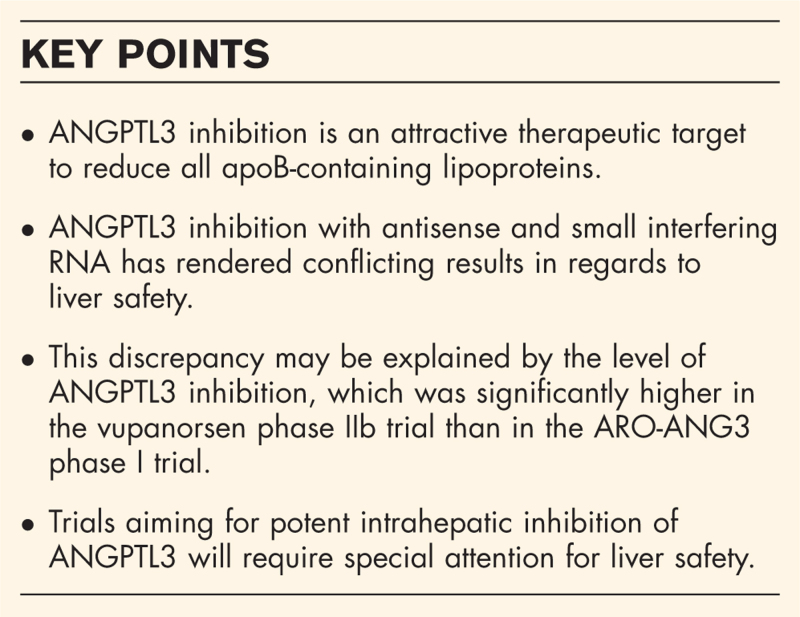
no caption available

### Background

In 1991, a form of familial hypobetalipoproteinaemia was reported, which was not due to a mutation in the apolipoprotein B or the microsomal triglyceride transfer protein gene [7]. Two decades later, Musunuru *et al*. [8] showed that a loss of function (LOF) mutation encoding for ANGPTL3 was the cause of this disorder [8]. The angiopoietin-like protein family consists of eight members, which are related to angiopoietins. Three members of this family have been demonstrated to have an impact on lipid metabolism: ANGPTL3, ANGPTL4 and ANGPTL8 [9]. ANGPTL3 is a protein secreted exclusively by the liver, and its expression is mainly regulated by the oxysterol-responsive liver-X receptor [10]. ANGPTL3 plays a pivotal role as a central orchestrator in triglyceride metabolism, acting as an inhibitor of both lipoprotein lipase (LPL) as well as endothelial lipase. Its inhibitory potency is dynamically modulated in a complex interplay with ANGPTL4 and ANGPTL8 with different roles during the fasting versus the postprandial state, which has been reviewed extensively elsewhere [11].

Currently, multiple genome-wide association studies have substantiated the association between LOF mutations in ANGPTL3 and a pan-hypocholesterolemia, characterized by a low concentration of triglyceride-rich lipoproteins, LDL-C as well as high-density-lipoprotein cholesterol (HDL-C). Homozygous carriers of functional variants in *ANGPTL3* were found to have an up to 71% lower triglyceride, 67% lower LDL-C, and 39% lower HDL-C compared with noncarriers [12], and carriers were at 40% reduced risk in ASCVD compared with noncarriers [13].

On the basis of these data, the interest in ANGPTL3 as a therapeutic target was sparked. Evinacumab, a mAb targeting plasma ANGPTL3, significantly reduced LDL-C and triglyceride levels by 47 and 55%, respectively, in patients with homozygous familial hypercholesterolemia (HoFH) [14]. On the basis of a potent LDL-C reduction of approximately 50% in both homozygous and heterozygous familial hypercholesterolemia (FH) patients, the Food Drug Administration as well as the European Medicines Agency approved this antibody for use in patients with HoFH [15]. Subsequent strategies applied the RNA-targeting platforms to selectively inhibit hepatic ANGPTL3 using antisense oligonucleotides (ASOs) or small interfering RNAs (siRNAs). Using vupanorsen, a N-acetylgalactosamine (GalNAc) antisense targeting hepatic ANGPTL3, the TRANSLATE study reported a significant liver transaminase increase resulting in discontinuation of this programme [16^▪▪^]. However, ARO-ANG3, a small interfering RNA (siRNA) targeting ANGPTL3, did not result in any adverse liver signals in a recently published phase I trial [17^▪▪^]. Here, we will discuss potential mechanisms and implications of these opposing findings.

### Therapies targeting ANGPTL3

#### Evinacumab

Although targeting circulating ANGPTL3 differs from RNA-based intracellular targeting strategies, we consider a brief overview of the liver data generated in the antibody studies to be pivotal. With respect to evinacumab (fully human mAb against ANGPTL3), a phase I trial in 83 healthy human volunteers demonstrated dose-dependent plasma lipid reductions in triglyceride and LDL-C up to 77 and 20%, respectively (Table 1) [18]. For seven participants (11.3%) in the evinacumab group, elevated alanine aminotransferase (ALT) levels were reported as an adverse event. The two participants with a transient increase of ALT higher than three times the upper limit of normal range (ULN) both had preexisting ALT elevation at baseline [18]. In the phase III trial in patients with HoFH, increased levels of either ALT or aspartate aminotransferase (AST) were reported in two of 44 participants (5%) in the treatment group versus two of 21 participants (10%) in the placebo group [14]. The transaminase levels during therapy did not exceed the three times ULN threshold. In the phase II trial for patients with refractory hypercholesterolemia, no liver safety concerns were reported [19^▪▪^]. More recently, evinacumab was administered in a group with markedly elevated triglyceride levels [20^▪▪^]. In these patients, the vast majority had an increased body mass index at baseline as well indications of fatty liver disease. A median 16% relative decrease in liver fat content after 12–24 weeks of evinacumab (15 mg/kg i.v.) administration was reported, without any notable changes in liver transaminases [20^▪▪^].

**Table 1:** Comparison of ANGPTL3 inhibition for efficacy and hepatic safety

Platform	Compound	Population	Efficacy (% change compared to placebo)	Liver safety in treatment group	Relative liver fat change from baseline
mAb	Evinacumab	*n* = 15: Multifactorial chylomicronemia syndrome + heterozygous LOF LPL Fasting TG: ≥500 mg/dl Hepatic fat fraction: 3--38%^a^	TG: -74% Non-HDL-C: -39% LDL-C: + 39%	AST/ALT > 3x ULN = 0%	-16%
Antisense oligonucleotide	Vupanorsen	*n* = 286 Fasting TG: 150–500 mg/dl Non-HDL-C: ≥100 mg/dl Hepatic fat fraction: 6--10%^b^	TG: -41% to -57% Non-HDL-C: -20% to -28% LDL-C: -8% to -16%	AST/ALT > 3x ULN = 44% (160 mg biweekly)	+ 76% (highest dose)
Small interfering RNA	ARO-ANG3	*n* = 203 Fasting TG: 150–500 mg/dl LDL-C ≥ 70 mg/dl or non-HDL-C ≥ 100 mg/dl^c^	TG: - 66% Non-HDL-C: - 41% LDL-C: - 37%	AST/ALT > 3x ULN = <1%	35 subjects with liver fat fraction >8% at baseline: - 30%

Adapted from [16^▪▪^,^20^^▪▪^,^22^].

#### Vupanorsen

Vupanorsen is a GalNAc conjugated antisense molecule inhibiting hepatic ANGPTL3. In the phase II trial of vupanorsen, 105 participants were included with fasting triglyceride more than 1.7 mmol/l, type 2 diabetes mellitus and hepatic steatosis (i.e. intrahepatic fat content > 8%) [21]. Participants were treated for 6 months with either placebo or vupanorsen. Administered dosages were 40 or 80 mg every 4 weeks, or 20 mg weekly. In individuals randomized to the 80 mg subcutaneous (SQ) every 4 weeks, ANPGPTL3 levels decreased by 59%, and triglyceride, LDL-C, non-HDL-C and apoB levels decreased by 53, 7, 18 and 9%, respectively, compared to placebo. This trial showed no clinically significant changes in AST, ALT or hepatic fat fraction.

In an effort to further evaluate the efficacy for non-HDL-C and total apoB lowering, the phase IIb TRANSLATE (Targeting ANGPTL3 with an Antisense Oligonucleotide in Adults with Dyslipidemia) trial was initiated in statin-treated adults with hyperlipidaemia, assessing the effect of dose-escalation of vupanorsen [16^▪▪^]. Two hundred and eighty-six participants were enrolled; 242 were randomly assigned to one of seven vupanoren dosing regimens, ranging in monthly equivalent doses of 80--320 mg, and 44 were randomized to placebo. The placebo-adjusted percentage change in non-HDL-C increased to -26.6% in the highest vupanorsen dose at 24 weeks coinciding with marked plasma ANGPTL3 reductions up to 95%. However, a dose-dependent increase in AST/ALT >3x ULN was observed in the vupanorsen group: zero participants in the 80 mg once every 4 weeks group, 2.3% (*n* = 1) in the 80 mg biweekly, 23.9% (*n* = 11) in the 120 mg biweekly and 44.4% (*n* = 16) of the participants in the 160 mg biweekly group. The transaminase increase was accompanied by a dose dependent relative increase in hepatic fat fraction of up to 76% in the 160 mg biweekly treatment group.

#### ARO-ANG3

ARO-ANG3 is a GalNAc-conjugated siRNA targeting ANGPTL3. In the phase I trial in 52 healthy volunteers with a fasting triglyceride more than 1.1 mmol/l [17^▪▪^], 24 individuals were exposed to a single ascending dose (35–300 mg subcutaneously), 12 to the multiple ascending dose (100–300 mg subcutaneously on days 1 and 29) and 16 comprised the placebo group. The follow-up was 10 weeks. A reduction in triglyceride and non-HDL-C of up to 58 and 26% was observed. Circulating ANGPTL3 levels were reduced by 63 to 84% (MAD group). One patient in the lowest 35 mg SAD cohort experienced a transient ALT elevation more than 3x ULN, and no AST increase more than 3x ULN was observed in the patients in the higher dose cohort. In a separate small cohort of nine individuals with liver fat more than 10% at baseline, six patients received 200 mg ARO-ANG3 at day 1 and 29 versus three in the placebo group. Liver fat fraction tended to decrease, with a mean relative change of -28% (ranging from +8 to −57%) at day 168 [17^▪▪^]. One patient who received ARO-ANG3 had a transient postdose peak increase in ALT more than 3x ULN.

Awaiting the full publication, interim results from the larger phase II study of ARO-ANG3 in adults with mixed dyslipidaemia and baseline liver fat content more than 10% (ARCHES-2, NCT04832971) were presented at the American Heart Association in 2022 [22]. Two hundred and three participants with mixed dyslipidaemia were included, with a fasting triglyceride between 150 and 499 mg/dl and either LDL-C at least 70 mg/dl or non-HDL-C at least 100 mg/dl; all were on stable statin therapy. Participants were randomized to 50, 100, 200 mg ARO-ANG3 or placebo at randomization and at week 12. At week 16, there was a dose-dependent mean reduction in ANGPTL3 of up to 71%, in triglyceride up to 59% and in LDL-C up to 32%. A dose-dependent relative reduction in liver fat fraction of approximately 30% was observed. One participant had a transient elevation of ALT more than 3x ULN; no other adverse events related to changes in liver function tests have been reported.

### Integrating hepatic ANGPTL3 inhibition data

In the quest to identify novel compounds able to reduce all apoB-containing lipoproteins in an LDL-C receptor independent manner, ANGPTL3 inhibition is clearly a promising candidate if adequate safety can be established. Whereas plasmatic inhibition of ANGPTL3 using monoclonal antibodies has proven well tolerated, RNA-targeting of ANGPTL3 in the liver yields heterogeneous findings. With antisense ANGPTL3 eliciting AST/ALT more than 3x ULN in 30–45% of individuals with a concomitant dose-dependent relative increase in hepatic fat fraction up to 75%, the silencing RNA has (preliminary) data reporting virtually no increase in transaminases with evidence of a dose-dependent relative decrease in hepatic fat fraction up to 30%. How can we reconcile these apparently discrepant findings?

A first clue may relate to the level of target inhibition. The liver transaminase signal emerged exclusively at the higher dosages vupanorsen of 120 and 160 mg biweekly, which induced a mean of 92--95% reduction in plasma ANGPTL3 [16^▪▪^]. In contrast, lower dosages vupanorsen with 70–85% as well as (repeated) dosing of ARO-ANG3 with 65–85% of plasma ANGPTL3 reduction were not associated with a significant liver transaminase signal [16^▪▪^,^17^^▪▪^]. In this scenario, maximal ANGPTL3 inhibition in the hepatocyte could potentially lead to liver fat accumulation due to reduced unloading of apoB-containing lipoproteins, as originally suggested by Musunuru *et al*. [8] reporting a gene-dose-dependent reduction of hepatic apoB secretion in subjects with hetero-/homozygous ANGPTL3 loss-of function mutations. It should be noted, however, that the role of ANGPTL3 on hepatic TRL excretion remains a matter of debate. On the one hand, a decreased hepatic VLDL excretion coinciding with increased hepatic triglyceride-synthesis was reported in ANGPTL3 knock-out mice [23]. In line, Xu *et al.*[24] substantiated that ANGPTL3 silencing by CRISPR-Cas decreased the secretion of apoB by more than 50% in various hepatic cell lines (Huh7 and HepG2), lending further support to a role of ANGPTL3 in enhancing hepatic apoB secretion. In contrast, obese mice treated with an ANGPTL3 targeting ASO had significant reductions in liver triglyceride secretion with a concomitant 81% lower liver triglyceride accumulation compared to control mice [25]. Human data also do not support a large impact of ANGPTL3 LOF and liver steatosis [26]. Whereas mendelian randomization studies have not shown an association between common genetic variants lowering hepatic expression of ANGPTL3 and transaminase levels or liver fat, this may also relate to the small effect sizes of these common variants on ANGPTL3 expression [27].

An alternative explanation may relate to off-target effects of vupanorsen, which has a molecule- and/or platform-specific origin. Historically, the apoB antisense mipomersen induced marked hepatic steatosis, but this was clearly an *on-target* effect of apoB inhibition [28]. Other antisenses have not been associated with hepatic steatosis, which does not support an overall off-target effect of the platform. A recent meta-analysis including data from six different GalNAc conjugated ASOs reported no significant liver transaminase signals following GalNAc ASO administration at dosages up to 320 mg/month [29^▪^].

Summarizing these partially controversial data, one can state that questions remain regarding the safety of (very) potent hepatic ANGPTL3 inhibition, as it might decrease hepatic unloading of apoB-containing lipoproteins, which subsequently give rise to hepatic fat accumulation. Unfortunately, since the efficacy of ANGPTL3 inhibition to lower total apoB-containing particles and non-HDL-C appears to be relatively limited, it is likely that (very) potent ANGPTL3-inhibitory strategies will have to be evaluated in patients with residually elevated triglyceride rich lipoproteins.

The solution to this unresolved question is likely to be provided by two emerging initiatives: First, the completion of the ARO-ANG3 studies in patients with increased apoB containing lipoprotein levels and fatty liver disease (data presentation planned at AHA 2023). A small caveat with respect to our unresolved ‘liver safety’ question is that ideally a dose-response curve with higher ARO-ANG3 dosages should be included aimed at achieving more than 90% plasma ANGPTL3 reduction. In the preliminary data, this dose was not included in the ARCHES-2 study [22]. Second, new data from the CRISPR-Cas ANGPTL3 programme have recently reported an approximately 96% reduction in plasma ANGPTL3 in nonhuman primates in absence of significant liver transaminase signals [30,31]. When this application will be evaluated in patients, we will be able to compare efficacy and safety of the predicted high-efficacy ANGPTL3 inhibition. However, it remains to be awaited if this treatment moiety will at first only be applied to patients with homozygous FH in absence of fatty liver disease.

## CONCLUSION

ANGPTL3 inhibition is an attractive therapeutic target to reduce all apoB-containing lipoproteins. For intrahepatic strategies, highly potent ANGPTL3 inhibition will for now require special attention for liver safety.

## Acknowledgements

*None.*

### Financial support and sponsorship

*E.S.G.S. was supported by the Netherlands Heart Foundation CVON 2017-20: generating the best evidence-based pharmaceutical targets for atherosclerosis [GENIUS II].*

*G.K.H. is employed part-time by Novo Nordisk and owns shares. R.F.O. has no disclosures.*

### Conflicts of interest

*There are no conflicts of interest.*

## REFERENCES AND RECOMMENDED READING

Papers of particular interest, published within the annual period of review, have been highlighted as:

▪ of special interest
▪▪ of outstanding interest
